# Gene set control analysis predicts hematopoietic control mechanisms from genome-wide transcription factor binding data

**DOI:** 10.1016/j.exphem.2012.11.008

**Published:** 2013-04

**Authors:** Anagha Joshi, Rebecca Hannah, Evangelia Diamanti, Berthold Göttgens

**Affiliations:** Department of Hematology, Cambridge Institute for Medical Research and Wellcome Trust and MRC Cambridge Stem Cell Institute, Cambridge University, Hills Road, Cambridge, UK

## Abstract

Transcription factors are key regulators of both normal and malignant hematopoiesis. Chromatin immunoprecipitation (ChIP) coupled with high-throughput sequencing (ChIP-Seq) has become the method of choice to interrogate the genome-wide effect of transcription factors. We have collected and integrated 142 publicly available ChIP-Seq datasets for both normal and leukemic murine blood cell types. In addition, we introduce the new bioinformatic tool Gene Set Control Analysis (GSCA). GSCA predicts likely upstream regulators for lists of genes based on statistical significance of binding event enrichment within the gene loci of a user-supplied gene set. We show that GSCA analysis of lineage-restricted gene sets reveals expected and previously unrecognized candidate upstream regulators. Moreover, application of GSCA to leukemic gene sets allowed us to predict the reactivation of blood stem cell control mechanisms as a likely contributor to LMO2 driven leukemia. It also allowed us to clarify the recent debate on the role of Myc in leukemia stem cell transcriptional programs. As a result, GSCA provides a valuable new addition to analyzing gene sets of interest, complementary to Gene Ontology and Gene Set Enrichment analyses. To facilitate access to the wider research community, we have implemented GSCA as a freely accessible web tool (http://bioinformatics.cscr.cam.ac.uk/GSCA/GSCA.html).

Cell type–specific gene expression is an inherent property of all multicellular organisms and indeed represents a major determinant that underlies the generation of differentiated cell types with distinct functionality. Elucidating the molecular mechanisms controlling cell type–specific expression has the power to reveal fundamental insights into the regulatory circuitry controlling both human and model organism development. Moreover, identification of control mechanisms in normal cells provides potential avenues for manipulating cellular fates, as exemplified by the recent explosion in cellular reprogramming studies [1]. It also enables the rational design of new therapies aiming to revert abnormal pathological cellular states back to their normal condition [1].

The blood or hematopoietic system has long been recognized as a powerful model system for studying cell type–specific gene expression [2]. Within the blood system, more than 10 distinct mature hematopoietic lineages (e.g., red blood cells, T cells, B cells) are generated from pluripotent hematopoietic stem cells (HSCs) via a sequence of intermediate progenitors, often represented as a lineage differentiation tree. Both the mature lineages as well as the various immature blood stem and progenitor populations can be purified based on the expression of combinations of specific cell surface markers, thus enabling powerful studies of cellular differentiation.

Transcription factors have long been recognized as major regulators of hematopoietic cell type specification [3–6]. To understand the mechanisms underlying cell type specification by transcription factors, it will be essential to identify their transcriptional targets. An important advancement in this research area was provided by the introduction of chromatin immunoprecipitation (ChIP) coupled to massively parallel sequencing (ChIP-Seq), which allows genome scale identification of all DNA sequences (regions) bound by a given transcription factor (TF) in a given cell type [7]. The technique has been rapidly adopted with over 100 individual studies now deposited in public databases for the murine hematopoietic system alone. This wealth of new data represents unprecedented opportunities to unravel the transcriptional control mechanisms that mediate expression of specific sets of genes within the various hematopoietic cell lineages [8].

Gene ontology [9] overrepresentation analysis provides information on various types of functional categories enriched within a given gene set of interest [10] and GSEA determines whether a gene set of interest shows statistically significant expression differences between two or more cell types [11]. However, neither of these approaches explicitly links a gene set to transcriptional control mechanisms. In this study, we report a new computational framework for linking gene sets with transcriptional control, called Gene Set Control Analysis (GSCA). Unlike previous algorithms developed to provide functional enrichment [10], GSCA links gene sets to likely upstream regulators responsible for coordinated expression. By exploiting multiple transcription factor binding patterns from genome-wide ChIP-Seq studies, GSCA can provide previously unattainable insights into possible transcriptional control mechanisms operating in both normal and malignant cells. To gain insights into combinatorial control mechanisms (i.e. multiple transcription factors occupying the same binding site in a gene locus), we further developed a novel tool called combinatorial-GSCA (C-GSCA). Through integrated analysis of 142 blood-specific ChIP-Seq binding datasets, C-GSCA identifies likely combinatorial transcriptional control mechanisms by revealing TF cooccupancy patterns specifically associated with gene regulatory elements from a given gene set. A web-based implementation of GSCA and C-GSCA allows user-friendly access for the wider research community, and thus provides a substantial new addition to the bioinformatic toolbox for hematopoietic gene set analysis.

## Methods

### ChIP-seq compendium

Binding events for 35 transcription factors in seven major hematopoietic lineages were obtained from Hannah et al. [8]. Sixty new ChIP datasets from 18 publications and ENCODE murine datasets were analyzed, starting from the raw data set in each case, and peaks were identified in each sample using the protocol described previously [8]. A supplementary website (http://bioinformatics.cscr.cam.ac.uk/BLOOD_compendium_PUBLISHED.html) lists the number of peaks, reference, and peak calling method for each of the ChIP dataset. All binding events were mapped to genes using the same protocol described previously [12]. Binding events in the promoter and gene body were associated to the corresponding gene, whereas intergene peaks were associated to the nearest gene on either side within 50 kb, such that each peak is assigned to at most two genes.

Tissue-specific enhancer elements in mouse were downloaded from [13] and *p* value was calculated for overlap between each of the 61 tissue-specific enhancer regions and blood-specific regulatory regions [8] using a hyper-geometric test (Supplementary Table 1, online only, available at www.exphem).

### GSCA method

Of 270,261 genomic regions bound by at least one TF (*N*), for a set of user-defined genes, we calculate the number of genomic regions mapped to the genes (*n*). For each ChIP-Seq ChIP dataset, the number of peaks (*m*) near user defined genes (*k*) is calculated. The *p* value is calculated using a hypergeometric test (Fischer exact test).

### cGSCA method

A matrix of binding events with 270,261 genomic regions as rows and overrepresented ChIP-seq data sets (*K*) from GSCA step as columns is generated. The ChIP-seq data sets (*K* columns) are then clustered using a hierarchic clustering with Pearson's correlation coefficient as a distance measure.

### Reference data set

Gene sets for 80 clusters of tightly coexpressed genes (their induction patterns) in 38 hematopoietic cell types were obtained from Novershtern et al. [14]. Human genes were mapped to orthologous mouse genes using MGI mammalian orthology (http://www.informatics.jax.org/orthology.shtml). We calculated the *p* value for each gene set with respect to each signature cluster using a hypergeometric test. We used the number of Novershtern clusters significantly overrepresented (Bonferroni corrected *p* < 0.001) for one or more transcription factor targets as a measure to evaluate performance while comparing different methods.

### Gene expression datasets

Nine gene expression signatures (d-erythroid, differentiated, d-lymphoid, d-myeloid, r-myelolymphoid, s-erythroid, s-mpp, s-myelolymphoid, and stem) were obtained from [15]. Differentially expressed genes in various leukemia datasets were downloaded from their respective publications. Gene lists were then interrogated against the ChIP-seq compendium using both GSCA and C-GSCA.

### GSCA web tool

The GSCA output was produced using R, and the web user interface of the application was done using Perl/CGI/HTML. R commands are executed through the perl–cgi script to produce the image. The web tool can be accessed at the following URL: http://bioinformatics.cscr.cam.ac.uk/GSCA/GSCA.html.

## Results

### Definition of a candidate regulatory genome in mouse hematopoiesis

We recently reported a compendium of more than 50 TF ChIP-Seq experiments in mouse blood cells collected from publicly available datasets [8]. We have doubled the compendium by adding 60 new ChIP datasets from 18 recently published studies [16–33] and ENCODE murine unpublished datasets to obtain genome-wide binding patterns for 53 unique transcription factors in 15 major blood lineages and three types of leukemia (Table 1). TF-bound peaks were determined for all new datasets using the same parameters as before [8], which resulted in a total of 270,261 genomic regions bound by at least one transcription factor. When added together, these 270,261 regions corresponded to 936 Mb, thus constituting 5.78% of the mouse genome. ChIP-Seq samples of the same transcription factor in related cell types were merged together to provide a consolidated set of 78 samples (Table 1).

Pennacchio et al. [13] developed a phylogenetic conservation and motif based approach to predict tissue specific enhancers, which allowed them to annotate ∼5,500 high-confidence mouse tissue-specific enhancers for 61 murine tissue types by integrating tissue-specific expression data, conservation information, and cis-regulatory motifs. Only 4 of these 61 tissues corresponded to hematopoietic cells, and predicted only enhancers for those four tissues showed significant overlap with our ChIP-enriched regions (B220^+^ B cells, *p* = 1.9e-10; CD4^+^ T cells, *p* = 1.4e-4; CD8^+^ T cells, *p* = 7.0e-7; lymph node, *p* = 1.0e-4; see Supplementary table 1). This analysis therefore supports the validity of a compendium built on TF binding events in hematopoietic cells.

### A new GSCA tool matches weighted TF-peak lists to gene sets

We next explored whether our blood-specific TF ChIP-Seq peak catalogue could be used to predict transcriptional control mechanisms that may regulate the coordinated expression of a given set of genes. Computational tools for the identification of statistically significant overlaps between a given gene set and peak regions from single ChIP-Seq experiments have been described previously [34,35]. However, these tools do not exploit the ever-increasing number of datasets for multiple TFs in the same or related cell types.

Novershtern et al. [14] reported gene expression profiles in 38 distinct purified populations of human hematopoietic cells ranging from hematopoietic stem cells, through multiple progenitor and intermediate maturation states, to 12 terminally differentiated cell types. Using the Module Networks algorithm [36], they identified 80 modules or gene sets of tightly coexpressed genes with distinct expression patterns and enrichment for specific biological functions, which they termed *induction patterns*. When we used the 80 Novershtern modules as gene sets, 37 of 80 gene sets (Supplementary Table 2, online only, available at www.exphem) showed a statistically significant correlation with one or more TF peak files from our compendium when using the previously described ChIP Enrichment Analysis (ChEA) [34] and Csan [35] tools. Of note, there was a good overlap between the cell type used for ChIP-Seq and the expression/induction patterns as annotated by Novershtern et al. (Supplementary Table 2). For example, gene set 727 with induction pattern “Late Erythroid” was associated with Eto2 in Erythroid, Scl, and Ldb1 in HSCs and Scl in MELs, and gene set 979 overrepresented for “immune response” genes with induction pattern “Late MYE” was associated with Cebpα, Cebpβ, P65, Pparg, and Stat1 in macrophages.

**Figure 1:**
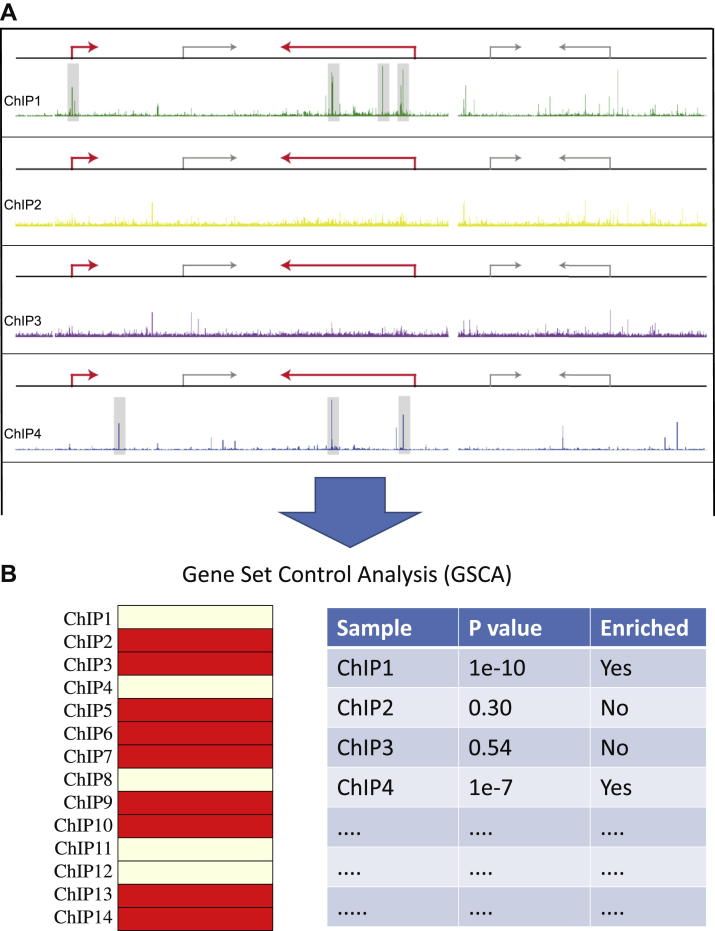
Schematic representation of the Gene Set Control Analysis (GSCA) protocol. For a given gene set of interest (red arrows), the number of peaks in gene loci is determined and a *p* value is calculated using a hypergeometric test. The TFs from overrepresented ChIP datasets (corrected *p* < 0.001, yellow bars in the figure) are then reported as candidate upstream transcriptional regulators. (For interpretation of the reference to color in this figure legend, the reader is referred to the web version of this article.)

Because the ChEA [34] and Csan [35] tools could associate candidate upstream regulators to less than half of the 80 Novershtern gene sets, we set out to develop an alternative approach by incorporating the concept of weighted peak-to-gene mapping recently reported as part of the Genomic Regions Enrichment of Annotations Tool (GREAT) [37]. GREAT links a list of ChIP-Seq peak regions to gene lists with particular functional significance and unlike previous approaches incorporates binding sites not only in the promoter region of a gene. Taking inspiration from this approach, we developed a new tool by mapping each peak to its nearest gene within 50 kb and then considering the number of binding events in each gene locus to calculate the significance of association between a gene locus and a given upstream regulator. (Essentially this is the reverse of GREAT, which associates peaks with genes, whereas our new procedure associates genes with peaks). Specifically, our new tool determines the number of binding events in the loci of genes of interest for each ChIP dataset (Fig. 1A, red arrows), and then calculates a *p* value using a simple hypergeometric test. Datasets with statistically significant overlaps (corrected *p* value cut-off <0.001) are then selected by interrogating all ChIP datasets independently against the gene list (Fig. 1B). When applied to the 80 gene modules from Novershtern et al. [14], our new tool reported significant associations with ChIP-Seq peaks for 65 gene modules (Supplementary Table 2, online only, available at www.exphem), which corresponds to 81% of all gene sets compared with only 46% using the previously reported ChEA and Cscan tools. Incorporation of weighted gene lists therefore results in a significant increase in the percentage of gene modules that can be linked to candidate upstream regulators. We named this new approach Gene Set Control Analysis, or GSCA. Only 61% of all Novershtern gene sets (49 of 80 gene modules) were enriched when the binding events only in promoters were selected, thus highlighting the likely importance of binding to nonpromoter regions, which compose 57% of all binding events in our datasets.

### GSCA correlates relevant combinations of transcription factors with hematopoietic gene sets

To investigate the potential biological relevance of the candidate upstream regulatory transcription factors matched with the 65 Novershtern gene sets by GSCA, we again used the induction patterns defined by Novershtern et al. as a measure of lineage-specific expression. The majority of gene sets (97%) showed good correspondence between the induction patterns and the cell types in which the TFs had been chipped (Supplementary Table 3, online only, available at www.exphem).

For example, gene sets 667 and 829 (enriched for T cell receptor activity) were associated by GSCA with Stats and Gata3 in T cells, whereas gene sets 649 and 961 (enriched for B cell receptor activity) were associated with Pu.1, E2A, and Pax5 in B cells. Gene set 721 (involved in inflammatory and antibacterial response) was linked by GSCA with Cebpα, Cebpβ, P65, Pu.1, and Stat1 in macrophages. Gene sets 727 and 889 with Late Ery induction pattern (enriched for protein amino acid glycosylation and blood group antigen functional annotations) significantly overlapped only with targets of Eto2, Gata2, Ldb1, Mtgr1, and Scl in erythroid cells. Taken together therefore, there is good concordance between the induction patterns of Novershtern gene sets and the matching ChIP-Sequencing TF datasets identified by GSCA.

### Combinatorial regulatory pattern discovery from multi factor ChIP-Seq data

**Figure 2:**
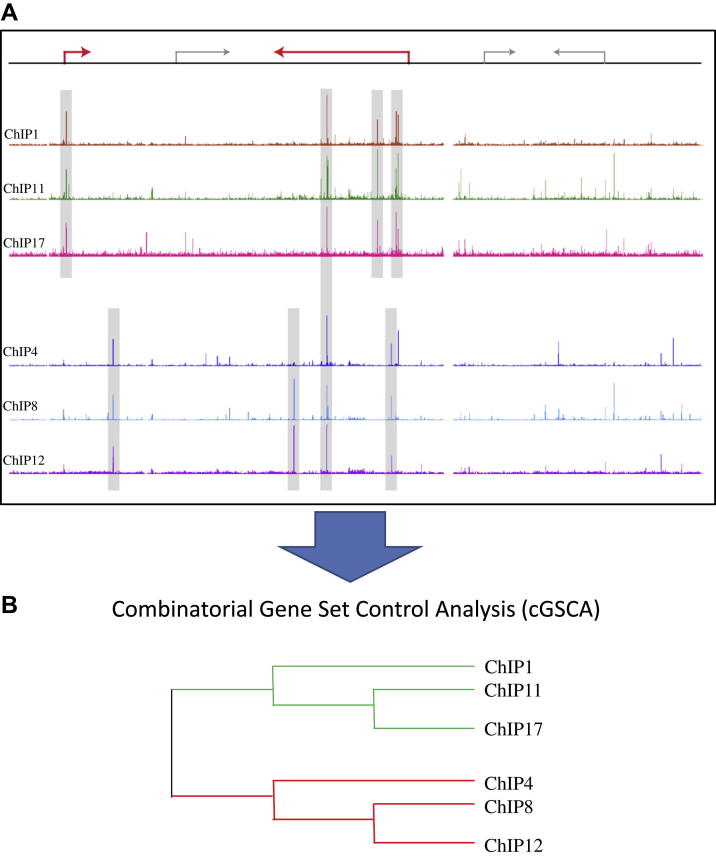
**(A)** Schematic representation of combinatorial Gene Set Control Analysis (cGSCA). A binary matrix of combinatorial binding patterns is generated using the overrepresented ChIP datasets from GSCA. **(B)** A hierarchical tree is then generated by clustering similar patterns. Color figure online.

Compared with previous tools, our new GSCA tool performs better by associating gene lists with ChIP-Seq peaks by calculating weighted associations between factors and genes based on the number of binding events within a gene locus. However, all individual ChIP-Seq datasets are treated independently, thus making it difficult to infer whether two overrepresented transcription factors work combinatorially (e.g. whether they show statistically significant co-occupancy of the same regulatory regions), rather than binding to overlapping sets of gene loci, but using distinct *cis*-regulatory regions. To address this issue of combinatorial binding, we developed a new tool called *combinatorial GSCA* (C-GSCA), and then applied this new tool to our hematopoietic ChIP-Seq compendium. For a given gene list, we first run GSCA to select the TFs showing overrepresented binding. Assuming that *m* TFs are selected out of 78 ChIP-seq datasets, we generate a binary matrix (*n* × *m*) of *m* columns representing the *m* ChIP datasets and *n* rows representing the genomic regions occupied by two or more of the *m* TFs, with 1s and 0s indicating the presence or absence of binding, respectively. We filter genomic regions bound by only one factor (∼16% of genomic regions; Supplementary Table 2, online only, available at www.exphem) because they are not informative in terms of combinatorial control mechanisms. We then perform hierarchical clustering of *n* overrepresented ChIP datasets using Pearson's correlation coefficient as a distance measure. Unlike GSCA, all overrepresented ChIP datasets are considered together, making the prediction of combinatorial control feasible (Fig. 2).

**Supplementary Figure 1:**
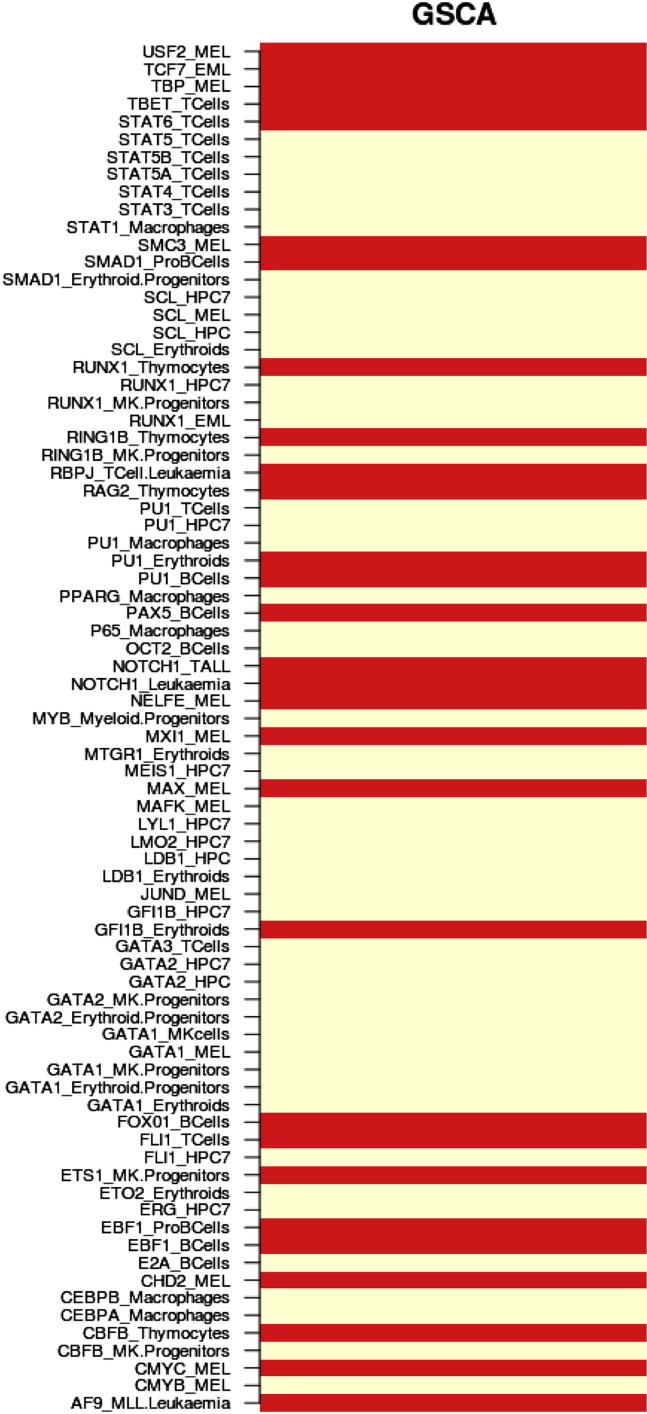
GSCA analysis of Heptad signature identified by Wilson et al. [12].

Using ChIP-Seq analysis of 10 transcription factors in the hematopoietic progenitor cell line HPC7, we have shown previously that combinatorial interactions between a heptad of TFs (SCL, LYL1, LMO2, GATA2, RUNX1, ERG, and FLI-1) were overrepresented in the loci of genes specifically expressed in HSPCs and therefore associated with gene sets specifically expressed in HSCs [12]. When the heptad-bound genes were interrogated using GSCA, 49 of 78 ChIP-Seq datasets were enriched, thus identifying multiple new transcription factors as candidate upstream regulators in addition to the seven factors (Supplementary Figure 1, online only, available at www.exphem). Using C-GSCA, these 49 datasets could be split into four cell type–specific groups of T cells, macrophages, HSCs, and erythroid (Supplementary Figure 1, online only, available at www.exphem). This observation suggests that gene loci bound by the heptad in blood stem and progenitor cells not only include genes specifically expressed in HSCs, but could also include a subset of genes affiliated with various different hematopoietic differentiation programs—an observation that would be consistent with the concept of lineage priming developed in the 1990s [38]. These results suggested that the C-GSCA procedure outlined here may be useful more generally to associate hematopoietic gene sets to upstream regulators and thus able to predict combinatorial control mechanisms driving the expression of a given gene set.

**Figure 3:**
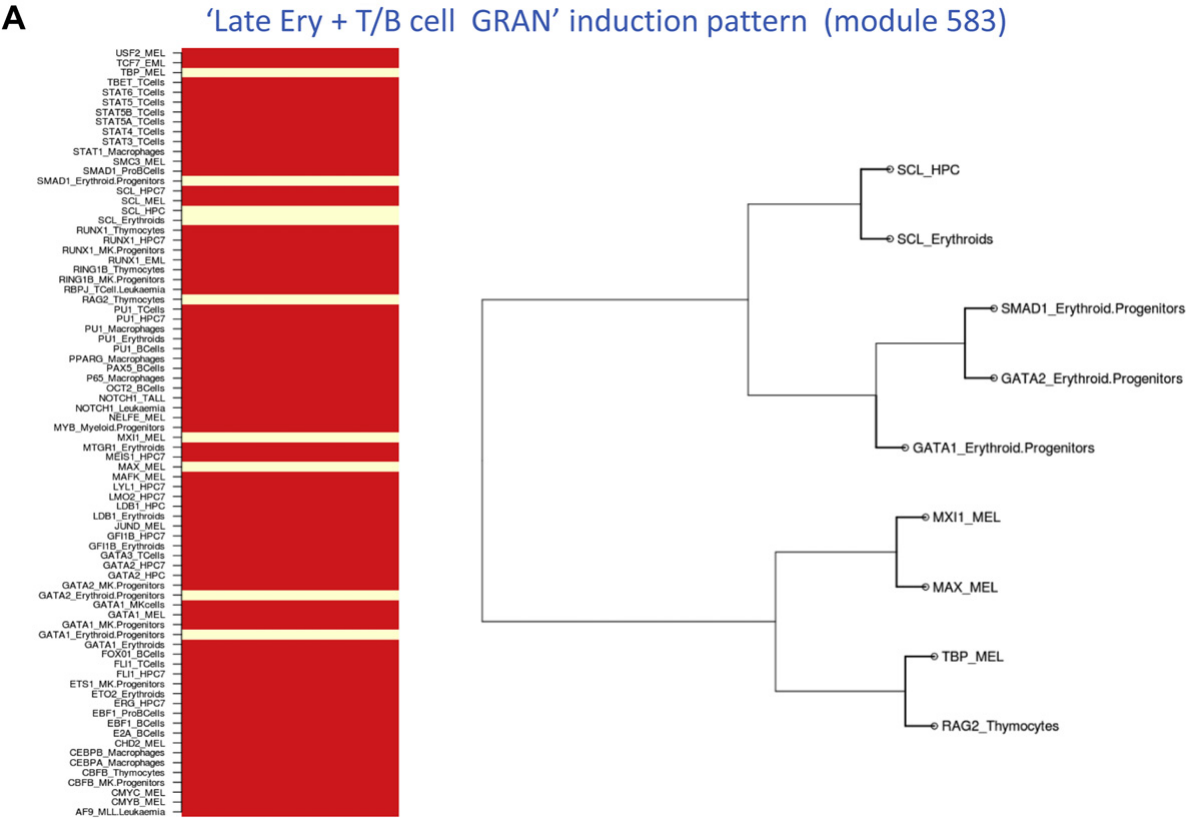

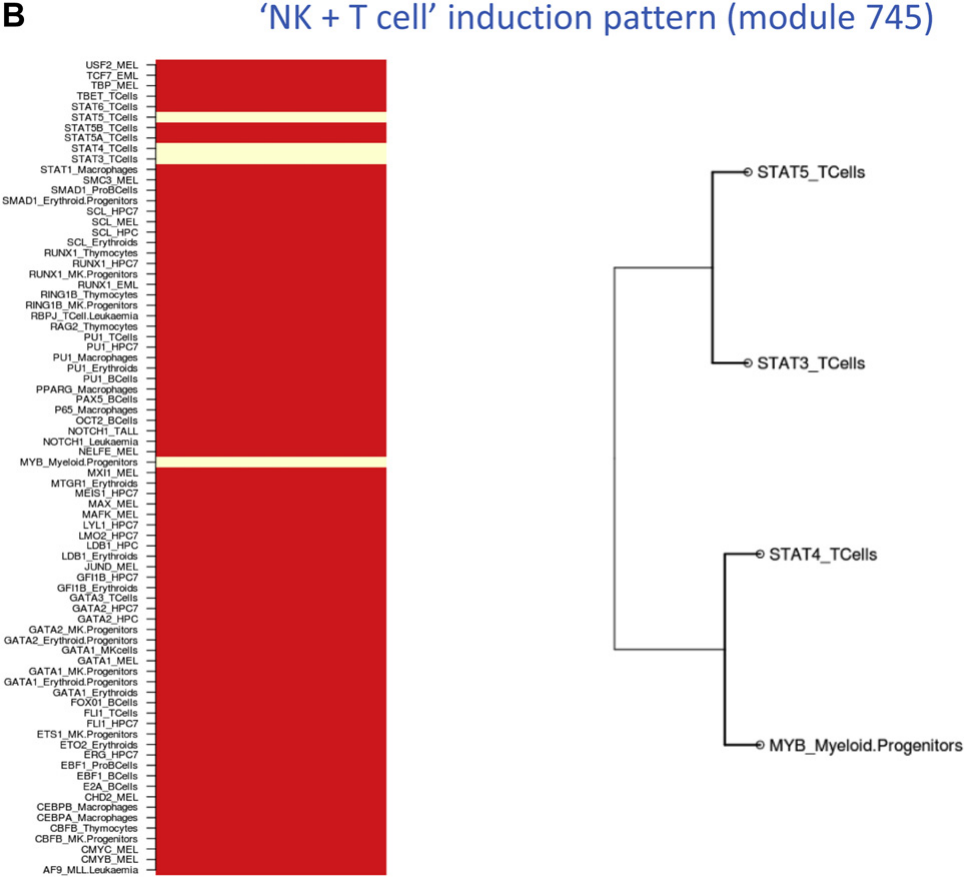
**(A)** Overrepresented regulators determined using GSCA (left) and C-GSCA (right) for gene module 583 from Novershtern et al. [14], with “Late Ery + T/B cells + GRAN” induction pattern. Unlike GSCA, C-GSCA can separate overrepresented independent binding patterns in different cell types (Gata1, Gata2, and Smad1 Erythroid progenitors and Max, Mxi1, and Tbp in MELs in this case). **(B)** Overrepresented regulators determined using GSCA (left) and C-GSCA (right) for gene module 745 from Novershtern et al. [14] with “NK + T cell” induction pattern. C-GSCA is able to separate combinatorial patterns in T cells and myeloid progenitors.

We next applied the new C-GSCA tool to all 80 hematopoietic gene sets from the Novershtern et al. study [14], which allowed us to associate 65 of the 80 Novershtern gene sets overrepresented for ChIP datasets using GSCA for combinatorial TF signatures. For example, Novershtern gene set 583 with induction pattern “Late Ery + T/B cell + GRAN” is associated with entirely different sets of transcription factors in two different cell types, because it was linked with Gata1, Gata2, Scl, and Smad1 in erythroid progenitors, and Rag2 in thymocytes, Max, Mxi1, and Tbp in mouse erythroleukemia (MEL) (Fig. 3A). Similarly, gene set 745 with induction pattern “NK + T cell” is linked with Myb in myeloid progenitors and Stat3, Stat4, and Stat5 in T cells (Fig. 3B). Indeed, more than 60% (40 of 65) of the overrepresented Novershtern gene sets with matched upstream regulators were linked with more than one combinatorial pattern (Supplementary Table 3, online only, available at www.exphem). Therefore, unlike the GSCA approach (Fig. 1), C-GSCA has the potential to identify distinct subsets of candidate upstream regulators for a given gene set (Fig. 2).

### GSCA web tool

**Figure 4:**
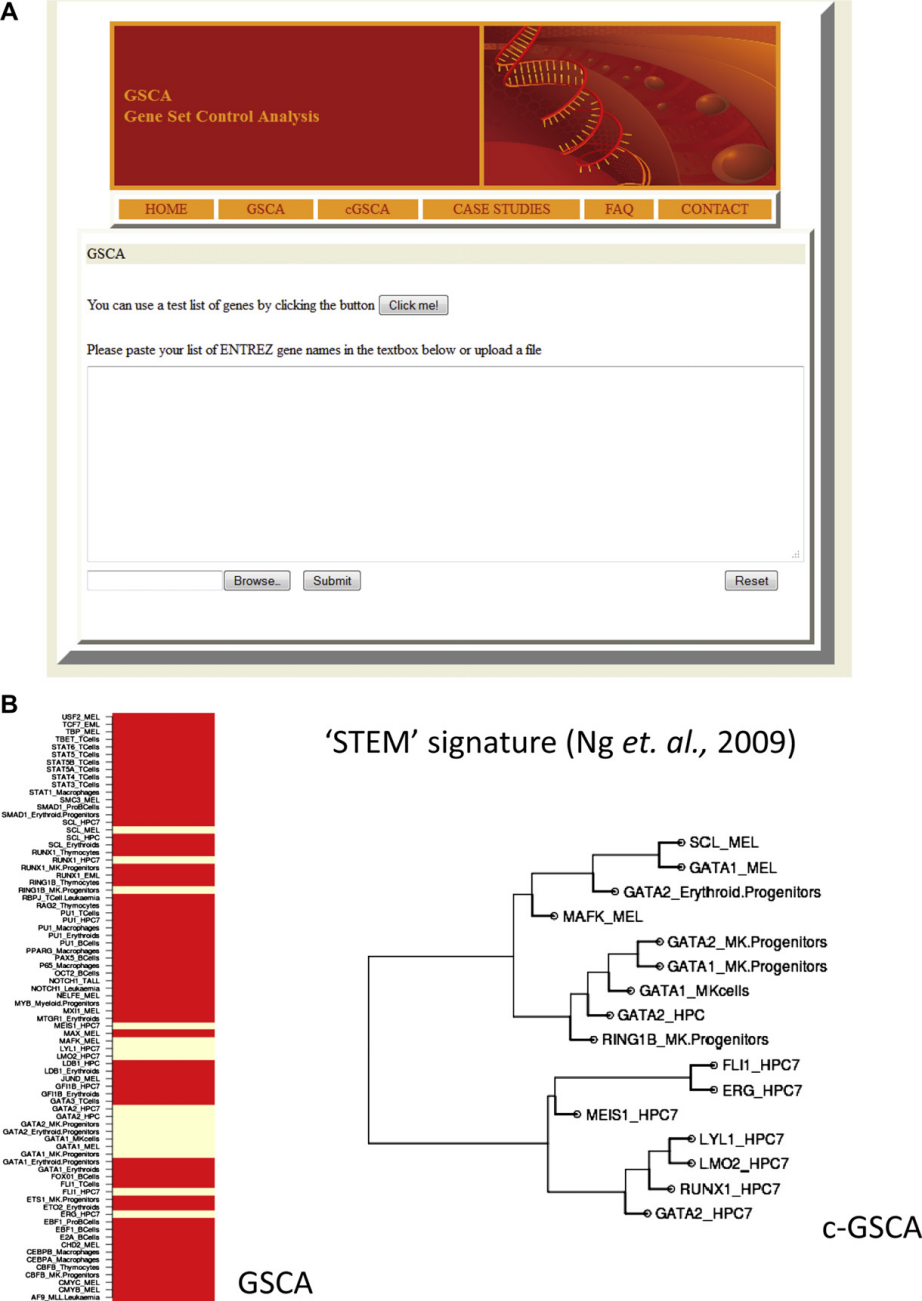
**(A)** Screen shot of Gene Set Control Analysis (GSCA) web tool with an option to either paste user defined gene list or upload from file, and to select method (GSCA or C-GSCA). **(B)** GSCA and C-GSCA output for stem signature dataset from Ng et al. [15] showing two cell type–specific distinct combinatorial patterns.

As GSCA and C-GSCA provide potentially powerful ways of predicting candidate upstream regulators for a given list, we developed a web tool to facilitate gene set control analysis for the wider community (http://bioinformatics.cscr.cam.ac.uk/GSCA/GSCA.html). In this section we provide a brief explanation of the functionality of the GSCA web tool using a recent transcriptome analysis of murine HSCs and early multipotent, bipotent, and unipotent progenitors [15], which reported nine gene expression signatures ranging from those characteristic for the most immature HSCs to those affiliated with differentiation into the individual hematopoietic lineages. We interrogated these nine experimentally obtained gene expression signatures using the GSCA web tool. Eight of these nine mouse stem–progenitor gene signatures showed significant overlap with multiple ChIP-Seq data sets, thus providing an independent test case to examine the biological relevance of predicted combinatorial regulatory signatures in addition to testing the functionality of the web tool (Supplementary Figure 3, online only, available at www.exphem). Figure 4A shows a screenshot of the web tool in which users can paste a query gene list or upload it from a file (human or mouse).

Upon choosing GSCA, a gene list of interest is interrogated against 78 ChIP-Seq datasets across 15 blood cell types. GSCA calculates the significance of overlap between each ChIP-Seq dataset and the gene set of interest and displays all ChIP-Seq datasets, with those showing enrichment in yellow color. For example, the self-renewing signature (*stem* signature from Ng et al. [15]) is provided as a test dataset for the users and shows statistically significant overlap with multiple transcription factors in HPC7 and progenitors. When the same *stem* signature gene list is analyzed using C-GSCA, the overrepresented ChIP datasets are clustered into two distinct cell type specific clusters HPC7 and MK progenitors (Fig. 4B). Six of the seven transcription factors in the HPC7 cluster overlap with the heptad signature—a binding pattern that we have previously shown is overrepresented in the loci of genes specifically expressed in HSPCs and therefore associated with gene sets specifically expressed in HSCs [12]. Similarly, the gene signature associated with the third wave of the myeloid lineage program (*d-my* signatures) from Ng et al. [15] shows statistically significant overlap with two combinatorial binding events, Cebpα, Cebpβ, Stat1, P65, and Pu.1 in macrophages and Myb in myeloid progenitors. In addition to showing the functionality of the web tool, these results suggest that combinatorial control signatures generated by C-GSCA have the potential to provide insights into combinatorial transcriptional control mechanisms, and that the GSCA web tool provides access to this type of analysis to the wider community.

### GSCA analysis of gene sets associated with hematologic malignancies

We have shown that GSCA can be used to link lineage-specific gene sets to combinations of candidate upstream regulatory TFs, and these associations are consistent with expectations based on current knowledge of regulatory control within hematopoiesis. This consistency attests to the potential robustness of the GSCA approach and suggests that it may also be useful to reveal biological insights into transcriptional programs operating in malignant hematopoietic cells, where diagnostic or prognostic gene sets have been derived for many types of leukemia, yet the combinations of TFs driving expression of these gene sets remain largely unknown. We therefore explored the utility of GSCA for linking leukemic gene sets with candidate upstream regulators.

We first analyzed a gene set recently reported by McCormack et al. [39], in which the investigators showed that overexpression of Lmo2 in T-lymphoid progenitors induced a preleukemic state characterized by extensive self-renewal capacity. When the authors performed comparative gene expression profiling of normal and LMO2 expressing thymocytes, they noted upregulation of several HSC specific genes and suggested that ectopic expression of Lmo2 might activate an HSC specific transcription program. To test this hypothesis further, we analyzed the list of genes upregulated in Lmo2 transgenic DN thymocytes [39] by GSCA. This analysis suggested that the LMO2 overexpression gene set was under the transcriptional control of stem cell transcription factors such as Scl, Gata2, Runx1, Fli1 and Erg and also showed a strong overlap with LMO2 binding itself in non-leukemic progenitor cells.

**Figure 5:**
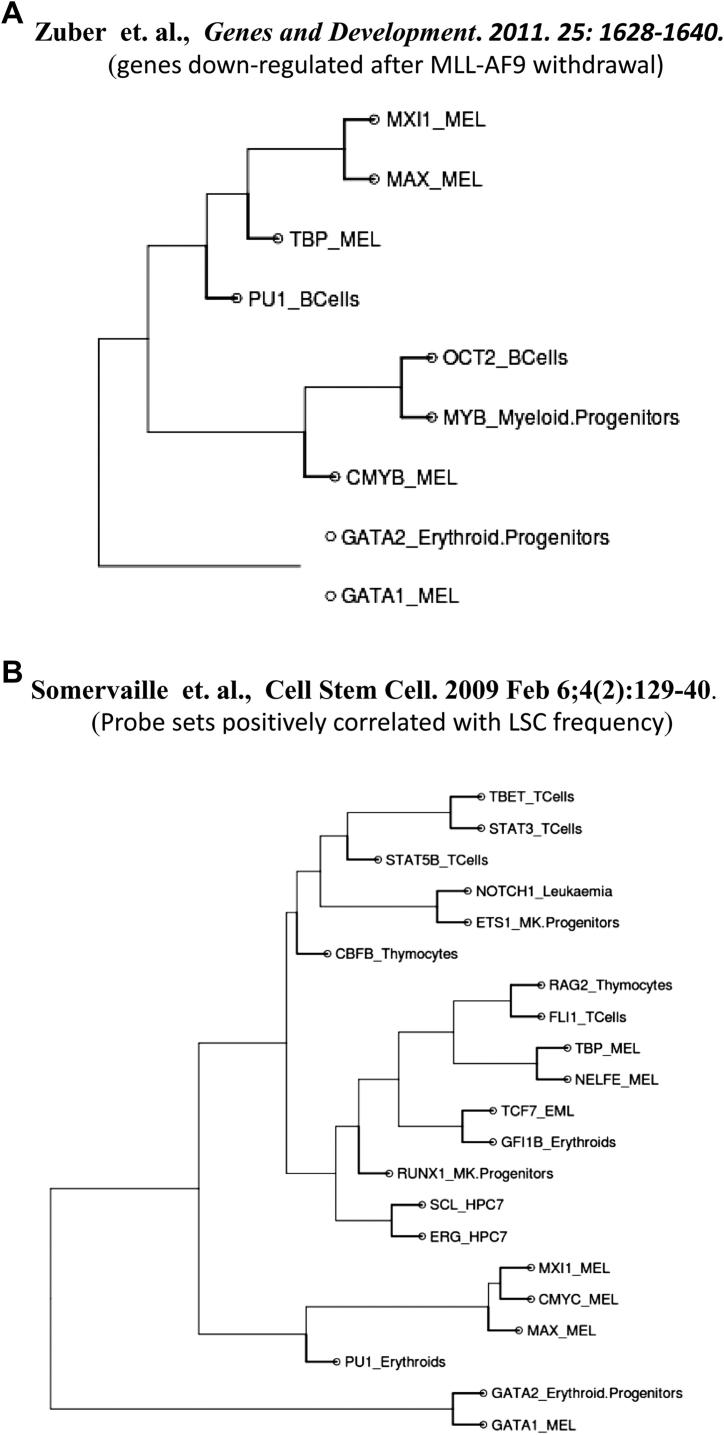
**(A)** Overrepresented regulators determined by C-GSCA for genes down regulated after MLL-AF9 withdrawal from Zuber et al. [40]. C-GSCA supports the notion that AF9 induces an Myb coordinated response. **(B)** Overrepresented regulators determined by C-GSCA for genes positively correlated with LSC frequency from Somervaille et al. [42]. C-GSCA identified cMyc and several other transcription factors to be overrepresented.

We next analyzed gene expression profiling data generated as part of a recent study investigating transcriptional programs downstream of mixed lineage leukemia (MLL) transformation in mouse models of acute myeloid leukemia (AML) [40]. Expression analyses following MLL-AF9 withdrawal had prompted the authors to propose a model whereby MLL-AF9 enforces a Myb-coordinated program of aberrant self-renewal that involves genes linked to leukemia stem cell potential and poor prognosis in human AML patients. Of note, when we analyzed the genes downregulated following MLL-AF9 withdrawal by GSCA, we observed statistically significant overlaps with the two Myb ChIP-Seq datasets in our compendium (Fig. 5A). In addition, GSCA also recovered associations with MAX and the MAX interacting protein MXI1, both of which have also been linked to a range of human cancers [41]. GSCA analysis therefore not only corroborated the findings by Zuber et al. [40]; it also provided additional hypotheses on likely mechanisms that might control transcriptional programs downstream of MLL-AF9 in AML.

**Supplementary Figure 2:**
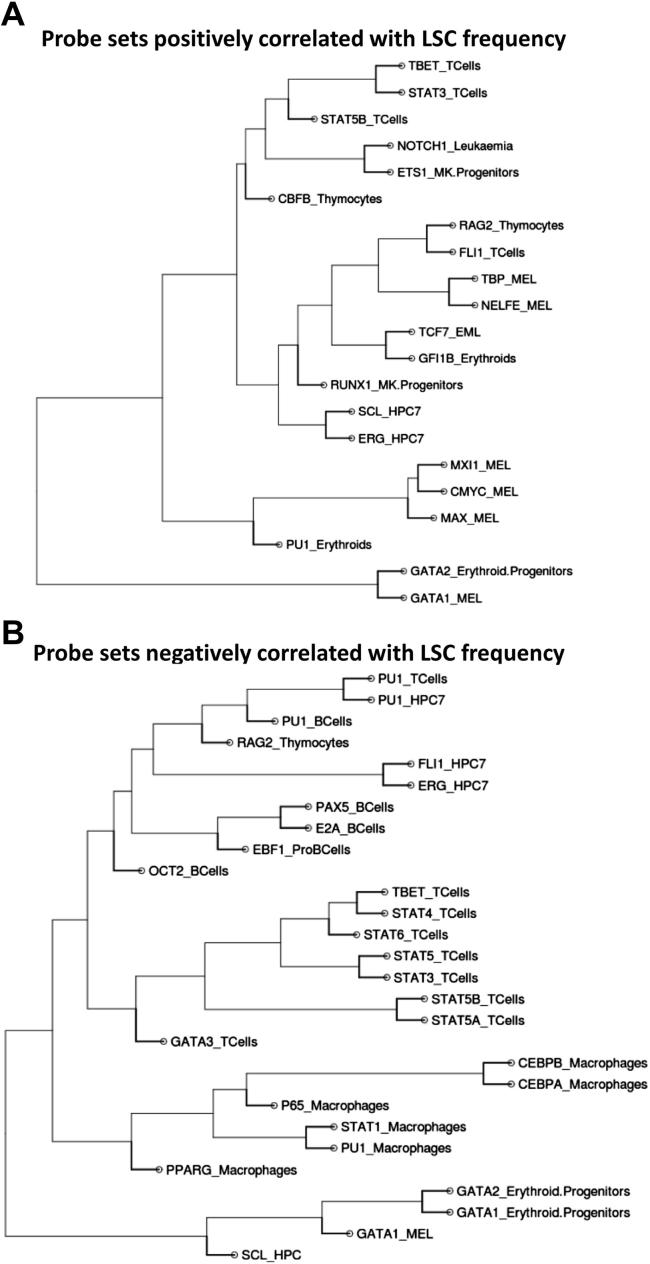
Hierarchical maintenance of MLL myeloid leukemia stem cells uses a transcriptional program shared with embryonic rather than adult stem cells [42]. **(A)** Probe sets positively correlated with LSC frequency. **(B)** Probe sets negatively correlated with leukemia stem cell (LSC) frequency.

**Supplementary Figure 3:**
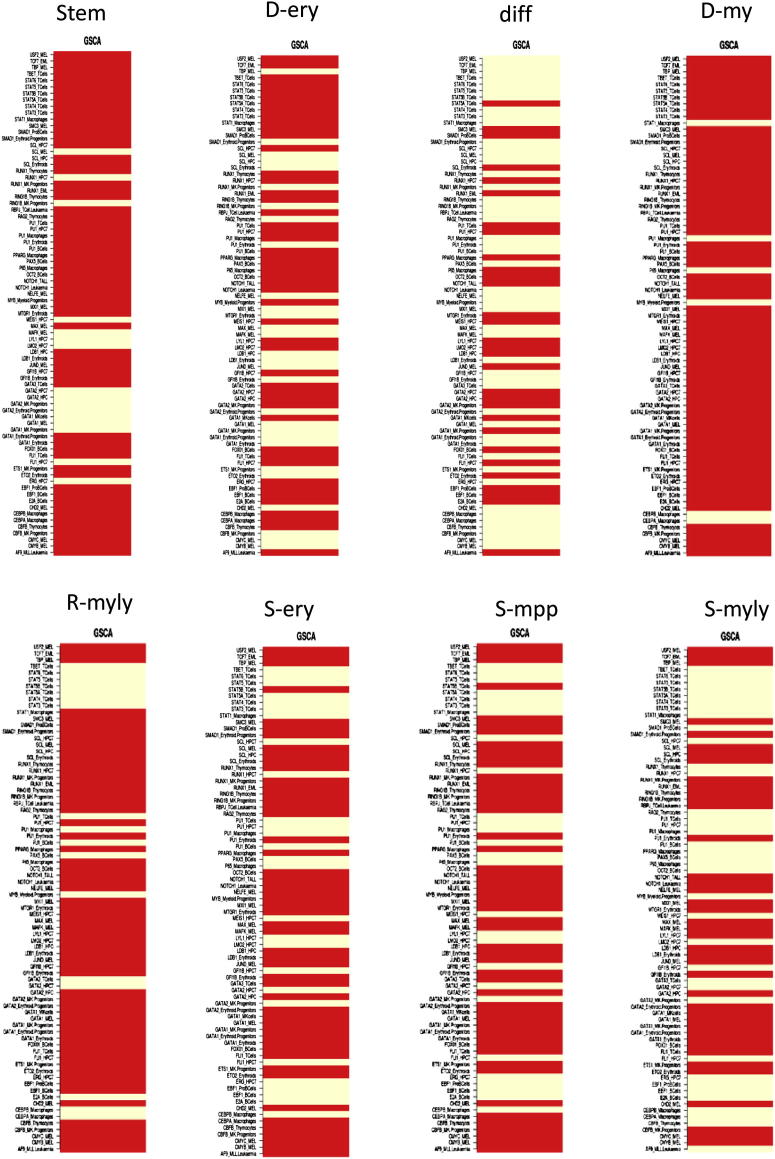
GSCA analysis of eight of nine gene expression signatures identified by Ng et al. [15].

The final leukemic gene set analyzed by GSCA was taken from a 2009 study of the transcriptional programs in leukemic stem cells [42]. Comprehensive gene expression profiling analysis had lead the authors to speculate that leukemia stem cells in an MLL-driven mouse model of AML are characterized by a transcriptional program shared with embryonic rather than adult stem cells. This conclusion was subsequently challenged when it was suggested that the overlap with embryonic stem cell transcriptional programs was the reflection for a shared dependence on c-MYC activity rather than related to the stemness phenotype of ES cells [43]. Analysis of the leukemia stem cell associated gene set from the Somervaille et al. [42] study by GSCA revealed a strong association with c-MYC ChIP-Seq datasets (Fig. 5B). However, there were also statistically significant associations with many additional ChIP-Seq datasets. GSCA analysis was therefore supportive of a role for c-MYC in the similarity between leukemic and embryonic stem cell expression signatures, but suggested that TFs more specifically expressed within blood cells also make important contributions to the leukemia stem cell transcriptional program. Of note, genes negatively associated with the leukemia stem cell phenotype in the study by Somervaille et al. [42] did not show the overlap with c-MYC, but it showed a distinct pattern of correlated ChIP-Seq datasets for the hematopoietic TFS, which interestingly contained several datasets for mature macrophages and was thus consistent with a relatively immature differentiation stage for the leukemia stem cells (Supplementary Figure 2, online only, available at www.exphem).

The application of GSCA to leukemic expression datasets supports the notion that integrated analysis of genome-wide transcription factor binding maps has significant potential as a new addition to the toolbox used by experimentalists to derive new hypothesis for experimental validation, which in the case of our current implementation of GSCA analysis would be geared specifically toward the identification of transcriptional mechanisms that control the behavior of normal and leukemic blood cells.

## Discussion

Gene expression arrays have been used widely to characterize genes responsible for a particular cellular phenotype. The differentially expressed genes thus obtained can then be used for functional enrichment analysis. However, the important question of “What upstream regulatory mechanisms are responsible for the differential expression?” is not specifically addressed when using current approaches for gene set analysis, such as Gene Ontology or Gene Se Enrichment analysis tools.

As a result of the rapid progress in next-generation sequencing technology, ChIP-Seq analysis has become a favorite tool to investigate in vivo binding events because it offers higher resolution, less noise, and greater coverage compared with other techniques [44]. Nevertheless, the generation of genome-wide binding maps for multiple transcription factors across different cell types remains a formidable challenge for individual labs [45]. ChIP-Seq datasets from different labs can, however, be integrated at the computational level, which we recently demonstrated using 53 mouse ChIP-Seq experiments from different laboratories across the hematopoietic differentiation tree [8]. Since then, we have added 60 new ChIP datasets, thus more than doubling the size of the original compendium. In addition to highlighting a potentially major portion of the total regulatory genome involved in hematopoietic gene expression, a data compendium of this scale should have the potential to provide new insights into regulatory mechanisms governing gene sets of interest.

To explore this further, we developed GSCA to identify enriched combinatorial binding patterns of transcription factors regulating a given gene set. This method uses experimental binding evidence, keeping the cell type specific context, unlike prediction methods based on overrepresentation of *cis*-regulatory sequence motifs in the promoters [46]. Using 80 clusters of tightly coexpressed genes in 38 hematopoietic cell types [14], we demonstrated that the transcriptional control mechanisms predicted are biologically coherent, and that GSCA performs better than current methods. Of note, this analysis also demonstrated that GSCA can be used in a cross-species fashion, with human gene sets analyzed using a murine ChIP-Seq compendium in this particular instance. The rationale for this cross-species capability is provided by recent observations from ChIP-Seq data for the same transcription factor in multiple species where it was shown that, although a significant proportion of binding locations (peaks) are not conserved, there tends to be what was termed *binding site turnover* for these sites where loss of binding in one species is accompanied by gains elsewhere in the same gene locus in the other species [47]. The conserved and many of the nonconserved binding sites therefore map to the same gene loci, such as in human–mouse comparisons. Just as for many other gene set analysis tools, cross-species capability in GSCA is facilitated by the use of standard gene symbols that are standardized across mammals.

We further illustrated the utility of the GSCA tool to unravel potential regulatory mechanisms underlying a range of leukemia gene sets, thus suggesting potential future application of GSCA to build hypotheses to investigate transcriptional control mechanisms responsible for the expression of gene sets with diagnostic, prognostic, or therapeutic relevance. Finally, we built a web tool to facilitate similar analysis for the wider scientific community. Complementary to gene ontology functional overrepresentation analysis, GSCA calculates overrepresentation of binding events for a gene list of interest, thus predicting possible transcriptional control mechanisms.

Given the significant investment into several collaborative projects such as the ENCODE (Encyclopaedia of DNA Elements) and modENCODE (model organism ENCODE) initiatives [48,49], we are likely to witness a near exponential increase in ChIP-Seq datasets over the coming years. Although our current implementation of the GSCA web tool is geared toward predicting candidate upstream regulators within hematopoietic cells, the approach can be applied easily to other tissues when sufficient ChIP-Seq data become available.

## Figures and Tables

**Supplementary Table 1:** The overlap between tissue specific enhancers identified by Pennacchio et al. [13] and the blood compendium showing that the enhancers in the compendium are highly blood specific

Tissue type	Number of enhancers	Overlap	*p* value
adipose tissue	213	86	0.99995
Adrenal gland	176	47	1
Amygdala	218	24	1
B220^+^ B cells	212	158	1.91E-10
Bladder	225	48	1
Blastocysts	191	63	1
Bone	200	87	0.99795
Bone marrow	224	101	0.99466
Brown fat	224	47	1
CD4^+^ T cells	226	148	0.000149
CD8^+^ T cells	194	137	7.00E-07
Cerebellum	180	24	1
Cerebral cortex	190	29	1
Digits	263	56	1
Dorsal root ganglia	193	45	1
Dorsal striatum	193	30	1
Embryo day 10	171	58	1
Embryo day 6	167	68	0.99961
Embryo day 7	163	51	1
Embryo day 8	170	44	1
Embryo day 9	174	63	1
Epidermis	292	58	1
Eye	255	32	1
Fertilized egg	176	33	1
Frontal cortex	197	21	1
Heart	227	62	1
Hippocampus	201	30	1
Hypothalamus	183	25	1
Kidney	230	43	1
Large intestine	208	62	1
Liver	267	27	1
Lung	241	75	1
Lymph node	245	160	0.000102
Mammary gland	198	33	1
Med	192	40	1
Olfactory bulb	194	32	1
Oocyte	173	37	1
Ovary	192	46	1
Pancreas	211	45	1
Pituitary	187	34	1
Placenta	202	59	1
Preoptic	176	29	1
Prostate	221	49	1
Salivary gland	213	46	1
Skeletal muscle	224	44	1
Small intestine	259	65	1
Snout epidermis	275	51	1
Spinal cord lower	197	39	1
Spinal cord upper	196	26	1
Spleen	228	108	0.97033
Stomach	206	46	1
Substantia nigra	183	29	1
Testis	197	31	1
Thymus	194	111	0.15904
Thyroid	239	47	1
Tongue	289	51	1
Trachea	250	72	1
Trigeminal	193	30	1
Umbilical cord	223	44	1
Uterus	181	58	1
Vomeralnasal organ	252	66	1

**Supplementary Table 2:** Thirty-seven gene sets of 80 with respective induction patterns from Novershtern et al. [14] found enriched using the method of Lachmann et al. [34] and Zambelli et al. [35]

Novershtern et al. clusters		Candidate upstream regulators	
#	Induction pattern	Transcription factor	Cell type
583	Late Ery + T/B cell + GRAN	TCF7 GFI1B SCL MAX, MXI1, NELFE, TBP ETS1 FLI1 RAG2	EML Erythroid HPC MEL MK progenitors T cells Thymocytes
607		TCF7 GFI1B, SCL P65, PPARG MXI1, NELFE MYB FLI1 RAG2, RING1B	EML HPC7 Macrophages MEL Myeloid progenitors T cells Thymocytes
649	B cell	E2A, EBF1, OCT2, PAX5, PU1 RUNX1 GFI1B, LDB1, MTGR1, PU1, SCL SCL FLI1, GATA2, MEIS1, PU1, SCL CEBPA, CEBPB, P65, STAT1 CMYB, CHD2, JUND, MAFK, MAX, MXI1, NELFE, SCL GATA1 CBFB, RING1B, RUNX1 AF9 MYB EBF1, SMAD1 RBPJ FLI1, GATA3, PU1, STAT3, STAT5A, STAT5B, STAT5, TBET CBFB, RAG2, RUNX1	B cells EML Erythroid HPC HPC7 Macrophages MEL MK cells MK progenitors MLL leukemia Myeloid progenitors Pro B cells T cell leukemia T cells Thymocytes
655	Mye	LDB1, SCL NELFE, SCL	HPC MEL
661	Late Ery + T/B – cell + GRAN	TCF7 NELFE FLI1 RAG2	EML MEL T Cells Thymocytes
667	T cell + NK	RUNX1 GATA2, RUNX1 RUNX1 GATA3, STAT5A, STAT5B CBFB, RUNX1	EML HPC HPC7 T Cells Thymocytes
673	T/B cell	E2A, EBF1, OCT2 GATA2, RUNX1, SCL CEBPA, CEBPB, P65, PPARG EBF1, SMAD1 FLI1, GATA3, PU1, STAT3, STAT4, STAT5A, STAT5B, STAT5, STAT6 CBFB, RING1B, RUNX1	B cells HPC7 Macrophages Pro B cells T cells Thymocytes
685	Early Mye + T/B cell + GRAN	RUNX1, TCF7 GFI1B, PU1 SCL CEBPA, CEBPB, P65 CMYC, MAX, MXI1, NELFE, TBP CBFB, ETS1, RUNX1 MYB FLI1, STAT4, STAT5B, STAT6, TBET CBFB, RAG2, RING1B, RUNX1	EML Erythroid HPC7 Macrophages MEL MK progenitors Myeloid progenitors T cells Thymocytes
703	T/B cell	RAG2	Thymocytes
715	Early Mye + T/B cell + GRAN	RAG2	Thymocytes
721	Late MYE + DCs	CEBPA, CEBPB, P65	Macrophages
727	Late Ery	ETO2 LDB1, SCL SCL	Erythroid HPC MEL
733	HSE + Early Mye	RUNX1, TCF7 GATA1, GATA2 GFI1B, MTGR1, SCL GATA2, GFI1B, LMO2, MEIS1, PU1, SCL CEBPA, CEBPB, P65, STAT1 GATA1, MAFK, MXI1, NELFE, TBP GATA1 GATA1, GATA2, RING1B MYB GATA3, STAT3, STAT5A, STAT5B, STAT5, STAT6, TBET RAG2, RING1B, RUNX1	EML Erythroid progenitors Erythroid HPC7 Macrophages MEL MK cells MK progenitors Myeloid progenitors T cells Thymocytes
739	Late Ery + T/B cell + GRAN	TCF7 MXI1, NELFE FLI1 RAG2	EML MEL T cells Thymocytes
763	Late MYE	EBF1 RUNX1, TCF7 PU1 FLI1, GFI1B, RUNX1, SCL CEBPA, CEBPB, P65, PPARG, STAT1 NELFE CBFB GATA3, STAT4, TBET RAG2	B cells EML Erythroid HPC7 Macrophages MEL MK progenitors T cells Thymocytes
793	Late Ery + T/B – cell + GRAN	TCF7 SCL NELFE ETS1, RUNX1 FLI1 CBFB, RAG2	EML HPC MEL MK progenitors T cells Thymocytes
799	NK + T cells (2)	E2A, FOX01, OCT2, PAX5, PU1 RUNX1 ETO2, PU1 GATA2, LDB1, SCL ERG, FLI1, GATA2, GFI1B, LMO2, LYL1, MEIS1, PU1, RUNX1, SCL CEBPA, CEBPB, P65, PPARG, PU1, STAT1 CMYB, CHD2, JUND GATA1 CBFB, GATA1, GATA2, RING1B MYB SMAD1 FLI1, GATA3, PU1, STAT3, STAT4, STAT5A, STAT5B, STAT5, STAT6, TBET CBFB, RAG2, RUNX1	B cells EML Erythroid HPC HPC7 Macrophages MEL MK cells MK progenitors Myeloid progenitors Pro B cells T cells Thymocytes
811	Early Mye + T/B cell + GRAN	TCF7 SCL CMYC, MXI1, NELFE, TBP, USF2 ETS1, RUNX1 FLI1 RAG2, RUNX1	EML HPC7 MEL MK progenitors T cells Thymocytes
817	T/B cell	E2A, EBF1, OCT2, PAX5 RUNX1, TCF7 ETO2, GFI1B, PU1, SCL GATA2, SCL ERG, FLI1, GFI1B, LMO2, MEIS1, PU1, RUNX1, SCL CEBPA, CEBPB, P65, PPARG, STAT1 CMYB, CHD2, MXI1, NELFE, SCL CBFB, GATA2, RING1B, RUNX1 EBF1, SMAD1 FLI1, GATA3, STAT3, STAT4, STAT5A, STAT5B CBFB, RAG2, RING1B, RUNX1	B cells EML Erythroid HPC HPC7 Macrophages MEL MK progenitors Pro B cells T cells Thymocytes
823	Early Mye + T/B cell + GRAN	MXI1, NELFE FLI1 RAG2	MEL T cells Thymocytes
835	Early Mye + T/B – cell + GRAN	GFI1B ERG CMYC, CHD2, MAX, NELFE, TBP ETS1, RUNX1 FLI1, STAT3, STAT6 RAG2	Erythroid HPC7 MEL MK progenitors T cells Thymocytes
841	Early Mye + T/B cell + GRAN	TCF7 SCL NOTCH1 CMYC, MAX, MXI1, NELFE, TBP ETS1 FLI1 RAG2	EML HPC7 Leukemia MEL MK progenitors T cells Thymocytes
859	T cell + NK	RING1B	Thymocytes
871	HSC + Early MYE	MXI1, NELFE FLI1 RAG2	MEL T cells Thymocytes
883	Late Ery + T/B cell + GRAN	PU1 TCF7 GATA1 GATA1, GFI1B ERG, SCL CEBPB CMYC, CHD2, MXI1, NELFE, TBP CBFB, RING1B, RUNX1 FLI1, STAT3, STAT4, STAT5, STAT6, TBET RAG2, RING1B, RUNX1	B cells EML Erythroid progenitors Erythroid HPC7 Macrophages MEL MK progenitors T cells Thymocytes
889	Late Ery	GATA1, GATA2, SMAD1 ETO2, GATA1, LDB1, MTGR1, SCL GATA2, LDB1, SCL LMO2, RUNX1 CEBPA, CEBPB CMYB, GATA1, MAFK, MAX, SCL, USF2 GATA1 GATA1, GATA2, RING1B, RUNX1	Erythroid progenitors Erythroid HPC HPC7 Macrophages MEL MK cells MK progenitors
901	Early Mye + T/B cell + GRAN	TCF7 GFI1B NOTCH1 CMYC, MXI1, NELFE, TBP ETS1 FLI1, STAT5B RAG2, RUNX1	EML Erythroid Leukemia MEL MK progenitors T cells Thymocytes
907	Late Ery + T/B cell + GRAN	TCF7 GATA1, GATA2 ETO2, GATA1, GFI1B, MTGR1 LDB1, SCL NOTCH1 CMYC, GATA1, MAX, MXI1, NELFE, SCL, TBP CBFB, ETS1, GATA1, RING1B FLI1, STAT3, STAT5B, STAT6, TBET RAG2	EML Erythroid progenitors Erythroid HPC Leukemia MEL MK progenitors T cells Thymocytes
925	Early Mye + T/B cell + GRAN	TCF7 CMYC, MXI1, NELFE, TBP FLI1, STAT6 RAG2	EML MEL T cells Thymocytes
943	T/B cell	TCF7 NELFE ETS1 FLI1 RAG2	EML MEL MK progenitors T cells Thymocytes
961	B cell	E2A, EBF1, OCT2	B cells
973	HSE + Early Mye	NELFE	MEL
979	Late MYE	CEBPA, CEBPB, P65, PPARG, STAT1 MYB	Macrophages Myeloid progenitors
985	Early Mye + T/B – cell + GRAN	CHD2	MEL
991	T/B cell	E2A, OCT2, PAX5, PU1 RUNX1, TCF7 GATA1, GATA2 GATA1, GFI1B, PU1 ERG, FLI1, GFI1B, MEIS1, PU1 CEBPA, CEBPB, P65, PPARG, STAT1 CMYC, CHD2, MAX, MXI1, NELFE, TBP CBFB, ETS1, RING1B, RUNX1 MYB EBF1 FLI1, PU1, STAT3, STAT4, STAT5A, STAT5B, STAT5, STAT6, TBET	B cells EML Erythroid progenitors Erythroid HPC7 Macrophages MEL MK progenitors Myeloid progenitors Pro B cells T cells Thymocytes
1003	Late Ery + T/B – cell + GRAN	NELFE RAG2	MEL Thymocytes
1021	Early Mye + T/B cell + GRAN	TCF7 GFI1B NELFE ETS1 FLI1 RAG2, RING1B, RUNX1	EML Erythroid MEL MK progenitors T cells Thymocytes

**Supplementary Table 3:** Sixty-five gene sets of 80 with respective induction patterns from Novershtern et al. [14] enriched for transcription factor binding regions across multiple blood tissues using GSCA: 63 of 65 show cell type and induction pattern matching

Novershtern et al. clusters		Combinatorial control signature	
No.	Induction pattern	Transcription factor	Cell type
399	None	STAT4, STAT5	T cells
559	NK + T cell (2)	STAT3, STAT4, STAT5A, STAT5B, STAT5, STAT6, TBET	T cells
571	Late MYE	CEBPA, CEBPB, P65, PU1, STAT1	Macrophages
583	Late ERY + T/B cell + Gran	GATA1, GATA2, SMAD1 SCL SCL MAX, MXI1, TBP RAG2	Erythroid progenitors Erythroid HPC MEL Thymocytes
607	Early MYE + T/B cell + Gran	PU1 ERG, FLI1, GFI1B, MEIS1, PU1, SCL CEBPA, CEBPB, P65, PPARG, PU1, STAT1 MYB GATA3, PU1, STAT3, STAT4, STAT5A, STAT5B, STAT5, STAT6, TBET	B cells HPC7 Macrophages Myeloid progenitors T cells
613	T/B – cell	PU1	B cells
619	Late MYE	CEBPA, CEBPB, PU1, STAT1	Macrophages
637	Late Ery	GATA1, GATA2, SMAD1 GATA1, LDB1, MTGR1, SCL ERG, LDB1 ERG GATA1, SCL GATA1, RING1B, RUNX1	Erythroid progenitors Erythroid HPC HPC7 MEL MK progenitors
643	HSE + Early Mye	GATA2	HPC7
649	B cells	E2A, PAX5, PU1 CEBPA, CEBPB, P65, PU1, STAT1	B cells Macrophages
655	Mye	GATA1, GATA2, SMAD1 GATA1, LDB1, MTGR1, SCL LDB1, SCL CEBPB, P65, PU1, STAT1 CMYB, CMYC, GATA1, MAFK, MXI1, SCL, TBP CBFB, GATA1, RING1B	Erythroid progenitors Erythroid HPC Macrophages MEL MK progenitors
661	Late Ery + T/B cell + GRAN	PU1 GATA1, GATA2 CMYB, CHD2, GATA1, MXI1, NELFE, TBP ETS1 FLI1 RAG2	B cells Erythroid progenitors MEL MK progenitors T cells Thymocytes
667	T cell + NK	GATA3, STAT3, STAT4, STAT5A, STAT5B, STAT5, STAT6, TBET CBFB, RAG2, RING1B, RUNX1	T cells Thymocytes
673	T/B cell	E2A, OCT2, PU1 CEBPB, P65, PU1, STAT1 GATA3, STAT3, STAT4, STAT5A, STAT5B, STAT5, STAT6, TBET	B cells Macrophages T cells
679	HSE + Early Mye	GATA2	Erythroid progenitors
685	Late MYE + T/B cell + GRAN	RUNX1, TCF7 GFI1B, PU1 ERG, PU1, SCL CEBPA, CEBPB, P65, PU1, STAT1 CMYC, GATA1, MXI1, NELFE, TBP CBFB, ETS1 FLI1, PU1 RAG2, RUNX1	EML Erythroid HPC7 Macrophages MEL MK progenitors T cells Thymocytes
703	T/B cell	TCF7 GFI1B ERG, PU1 NOTCH1 CMYC, MAX, MXI1, NELFE, TBP CBFB, ETS1 FLI1, STAT3, STAT4, STAT5B, STAT6, TBET CBFB, RAG2	EML Erythroid HPC7 Leukemia MEL MK progenitors T cells Thymocytes
709	General mild induction	ETO2 AF9	Erythroid MLL leukemia
715	Early MYE + T/B cell + GRAN	PU1 TCF7 GFI1B CEBPA, CEBPB, PU1, STAT1 CMYC, GATA1, MXI1, NELFE, TBP ETS1 FLI1 RAG2	B cells EML Erythroid Macrophages MEL MK progenitors T cells Thymocytes
721	Late MYE + DCs	CEBPA, CEBPB, P65, PU1, STAT1 MYB	Macrophages Myeloid progenitors
727	Late Ery	GATA1, GATA2, SMAD1 ETO2, GATA1, GFI1B, LDB1, MTGR1, SCL LDB1, SCL CMYC, GATA1, MAFK, MAX, MXI1, SCL, TBP CBFB, GATA1, GATA2, RING1B, RUNX1	Erythroid progenitors Erythroid HPC MEL MK progenitors
733	HSC + Early MYE	CEBPA, CEBPB, P65, PU1, STAT1 MYB	Macrophages Myeloid progenitors
739	Late ERY + T/B cell + Gran	PU1 TCF7 GATA1, GATA2 GFI1B, PU1 ERG, PU1 NOTCH1 CMYC, CHD2, GATA1, MAX, MXI1, NELFE, TBP CBFB, ETS1, RUNX1 FLI1 RAG2, RUNX1	B cells EML Erythroid progenitors Erythroid HPC7 Leukemia MEL MK progenitors T cells Thymocytes
745	General mild induction	MYB STAT3, STAT4, STAT5	Myeloid progenitors T cells
757	T cell + NK	TCF7 CMYC, MAX, MXI1, NELFE, TBP FLI1, STAT3, STAT5, TBET RAG2	EML MEL T cells Thymocytes
763	Late MYE	ERG, FLI1 CEBPA, CEBPB, P65, PPARG, PU1, STAT1 PU1, STAT3, STAT4, STAT5, TBET	HPC7 Macrophages T cells
769	T/B cell	PU1 CEBPA, CEBPB GATA3, STAT3, STAT4, STAT5A, STAT5B, STAT5, STAT6, TBET	B cells Macrophages T cells
775	Mye	GATA1	MK cells
781	General mild induction	NOTCH1	TALL
787	MYE	SMAD1 CEBPA, CEBPB, STAT1 GATA1, MAFK	Erythroid progenitors Macrophages MEL
793	Late ERY + T/B cell + Gran	PAX5, PU1 TCF7 GATA1, GATA2, SMAD1 GATA1, PU1, SCL PU1, SCL PU1 CEBPA, CEBPB, PU1, STAT1 CMYB, CMYC, GATA1, MAX, MXI1, NELFE, SCL, TBP GATA1 CBFB, ETS1, RING1B, RUNX1 FLI1, GATA3, STAT3, STAT4, STAT5A, STAT5B, STAT5, STAT6, TBET CBFB, RAG2, RUNX1	B cells EML Erythroid progenitors Erythroid HPC HPC7 Macrophages MEL MK cells MK progenitors T cells Thymocytes
799	NK + T cell (2)	E2A CEBPA, CEBPB, P65, PU1, STAT1 GATA3, PU1, STAT3, STAT4, STAT5A, STAT5B, STAT5, STAT6, TBET	B cells Macrophages T cells
805	HSE + Early Mye	ETO2	Erythroid
811	Late MYE + T/B cell + GRAN	TCF7 GFI1B ERG, SCL CEBPA, CEBPB, PU1 CMYC, CHD2, MAX, MXI1, NELFE, TBP, USF2 CBFB, ETS1, RUNX1 FLI1, STAT4, STAT5B, STAT5, STAT6, TBET CBFB, RAG2, RING1B, RUNX1	EML Erythroid HPC7 Macrophages MEL MK progenitors T cells Thymocytes
817	T/B cell	E2A, EBF1, PAX5, PU1 ERG, MEIS1, PU1 CEBPA, CEBPB, P65, PPARG, PU1, STAT1 RING1B EBF1, SMAD1 FLI1, PU1, STAT3, STAT4, STAT5A, STAT5B, STAT5, STAT6, TBET CBFB, RAG2, RUNX1	B cells HPC7 Macrophages MK progenitors Pro B cells T cells Thymocytes
823	Early MYE + T/B cell + GRAN	TCF7 GATA2 GFI1B SCL NOTCH1 CMYC, CHD2, MAX, MXI1, NELFE, TBP CBFB, ETS1 RBPJ FLI1, STAT3, STAT6 RAG2	EML Erythroid progenitors Erythroid HPC7 Leukemia MEL MK progenitors T cell leukemia T cells Thymocytes
829	T cell + NK	E2A GATA3, STAT3, STAT4, STAT5A, STAT5B, STAT5, STAT6, TBET CBFB, RUNX1	B cells T cells Thymocytes
835	Early MYE + T/B cell + GRAN	PAX5 TCF7 GFI1B ERG NOTCH1 CMYC, CHD2, MAX, MXI1, NELFE, TBP CBFB, ETS1, RUNX1 RBPJ FLI1 RAG2	B cells EML Erythroid HPC7 Leukemia MEL MK progenitors T cell leukemia T cells Thymocytes
841	Early MYE + T/B cell + GRAN	PU1 TCF7 GFI1B, PU1 ERG, GFI1B, PU1, SCL NOTCH1 PU1 CMYC, CHD2, MAX, MXI1, NELFE, TBP ETS1, RUNX1 AF9 RBPJ FLI1, PU1, STAT3, STAT4, STAT5B, STAT6, TBET RAG2	B cells EML Erythroid HPC7 Leukemia Macrophages MEL MK progenitors MLL leukemia T cell leukemia T cells Thymocytes
847	Late Ery + T/B cell + GRAN	PAX5 TCF7 GATA1, GFI1B GFI1B NOTCH1 CMYC, CHD2, MAX, MXI1, NELFE, TBP CBFB, ETS1, RUNX1 RBPJ FLI1, STAT3, STAT4, STAT5B, STAT6, TBET RAG2	B cells EML Erythroid HPC7 Leukemia MEL MK progenitors T cell leukemia T cells Thymocytes
853	Late MYE	PU1 ERG, PU1 CEBPA, CEBPB, P65, PU1, STAT1 PU1, STAT3, STAT4, STAT5A, STAT5, STAT6	B cells HPC7 Macrophages T cells
859	T cell + NK	GATA3, STAT3, STAT5, TBET	T cells
865	HSE + early Mye	GATA2, SMAD1 GATA1	Erythroid progenitors MEL
871	HSE + Early MYE	TCF7 GFI1B ERG, MEIS1, PU1 NOTCH1 CEBPA, CEBPB, PU1, STAT1 CMYC, MAX, MXI1, NELFE, TBP CBFB FLI1, STAT3, STAT6, TBET RAG2	EML Erythroid HPC7 Leukemia Macrophages MEL MK progenitors T cells Thymocytes
883	Late MYE + T/B cell + Gran	TCF7 GATA1, GFI1B SCL, SCL SCL CMYC, CHD2, MXI1, NELFE, TBP RING1B, RUNX1 FLI1, STAT4, STAT6, TBET RAG2, RUNX1	EML Erythroid HPC HPC7 MEL MK progenitors T cells Thymocytes
889	Late ERY	GATA1, GATA2, SMAD1 ETO2, GATA1, LDB1, MTGR1, SCL LDB1, SCL GATA1, SCL GATA1, GATA2, RING1B STAT4	Erythroid progenitors Erythroid HPC MEL MK progenitors T cells
895	Late ERY	GATA2 GATA1, LDB1 CEBPA, CEBPB, PU1, STAT1 MXI1	Erythroid progenitors Erythroid Macrophages MEL
901	Late MYE + T/B cell + Gran	TCF7 GATA1, GATA2, SMAD1 GFI1B, PU1 GFI1B NOTCH1 CMYC, GATA1, MAX, MXI1, NELFE, TBP CBFB, ETS1 FLI1, STAT3, STAT5B, STAT5 CBFB, RAG2, RING1B, RUNX1	EML Erythroid progenitors Erythroid HPC7 Leukemia MEL MK progenitors T cells Thymocytes
907	Late ERY + T/B cell + Gran	PAX5, PU1 TCF7 GATA1, GATA2, SMAD1 ETO2, GATA1, GFI1B, LDB1, MTGR1, SCL ERG, LDB1, SCL ERG NOTCH1 CMYB, CMYC, CHD2, GATA1, MAX, MXI1, NELFE, SCL, TBP CBFB, ETS1, GATA1, RING1B, RUNX1 FLI1 RAG2	B cells EML Erythroid progenitors Erythroid HPC HPC7 Leukemia MEL MK progenitors T cells Thymocytes
919	HSE + Early Mye	FOX01 ERG CMYC, MXI1, NELFE, TBP	B cells HPC7 MEL
925	Early MYE + T/B cell + GRAN	PAX5, PU1 TCF7 GATA1, GATA2, SMAD1 GATA1, GFI1B, PU1 ERG, SCL, SCL ERG, SCL CMYC, CHD2, GATA1, MAX, MXI1, NELFE, SCL, TBP CBFB, ETS1, RING1B, RUNX1 FLI1, STAT3, STAT4, STAT5B, STAT5, STAT6, TBET RAG2, RING1B	B cells EML Erythroid progenitors Erythroid HPC HPC7 MEL MK progenitors T cells Thymocytes
931	None	GATA1 STAT4, STAT5B, STAT5	MEL T cells
943	T/B cell	TCF7 GATA2 PU1 CMYC, MXI1, NELFE, TBP ETS1 FLI1, GATA3, STAT3, STAT4, STAT5B, STAT5, STAT6, TBET CBFB, RAG2, RUNX1	EML Erythroid progenitors Erythroid MEL MK progenitors T cells Thymocytes
949	T/B cell	GATA2 RAG2	Erythroid progenitors Thymocytes
955	T cell + NK	GATA3, STAT3, STAT4, STAT5A, STAT5B, STAT5, STAT6, TBET	T cells
961	B cell	E2A, EBF1, OCT2, PAX5, PU1 CEBPA, CEBPB, P65, PU1, STAT1	B cells Macrophages
967	Late ERY + T/B cell + Gran	PAX5 TCF7 GATA1, GFI1B, LDB1 ERG, FLI1, MEIS1, PU1, SCL NOTCH1 CMYC, CHD2, MAX, MXI1, NELFE, SMC3, TBP CBFB, ETS1, RING1B, RUNX1 FLI1, STAT4, STAT6, TBET RAG2	B cells EML Erythroid HPC7 Leukemia MEL MK progenitors T cells Thymocytes
973	HSE + Early Mye	CMYC, GATA1, MAX, MXI1	MEL
979	Late MYE	PU1 GFI1B CEBPA, CEBPB, P65, PPARG, PU1, STAT1 EBF1 STAT3, STAT4, STAT5, STAT6	B cells HPC7 Macrophages Pro B cells T cells
985	Early MYE + T/B cell + Gran	TCF7 GFI1B ERG, SCL CMYC, CHD2, MAX, MXI1, NELFE, TBP ETS1, RUNX1 FLI1, STAT3, STAT4, STAT6, TBET CBFB, RAG2, RUNX1	EML Erythroid HPC7 MEL MK progenitors T cells Thymocytes
991	T/B cell	PU1 GATA2 PU1 ERG CEBPA, CEBPB, PU1, STAT1 MXI1, TBP FLI1, STAT3, STAT4, STAT5B, STAT5, STAT6, TBET RAG2	B cells Erythroid progenitors Erythroid HPC7 Macrophages MEL T cells Thymocytes
997	NK + T cell (2)	STAT4	T cells
1003	Late ERY + T/B cell + Gran	TCF7 GATA1, GFI1B, PU1, SCL ERG, PU1, SCL ERG, PU1 NOTCH1 CMYC, CHD2, MAX, MXI1, NELFE, TBP CBFB, ETS1, RING1B, RUNX1 RBPJ FLI1, GATA3, STAT3, STAT6 CBFB, RAG2, RING1B, RUNX1	EML Erythroid HPC HPC7 Leukemia MEL MK progenitors T cell leukemia T cells Thymocytes
1009	HSE + Early Mye	CEBPA, CEBPB, P65, PU1, STAT1	Macrophages
1021	Early MYE + T/B cell + GRAN	PAX5 TCF7 GATA1 GFI1B, PU1 SCL NOTCH1 CMYC, CHD2, MXI1, NELFE, TBP ETS1, RUNX1 FLI1 RAG2, RING1B, RUNX1	B cells EML Erythroid progenitors Erythroid HPC7 Leukemia MEL MK progenitors T cells Thymocytes

**Table 1:** Seventy-eight ChIP-Seq binding peak files covering 53 unique transcription factors in 15 major blood lineages

Cell type	Transcription factors
Lymphocytes	
B cells	E2A, Ebf, Foxo1, Oct2, Pax5, Pu.1
T cells	Gata3, Fli1, Pu.1, Stat3, Stat4, Stat5, Stat5a, Stat5b, Stat6, Tbet
Thymocytes	Cbfb, Rag2, Ring1b, Runx1
Progenitors	
HPC	Gata2, Ldb1, Scl
HPC7	Erg, Fli1, Gata2, Gfi1b, Lmo2, Meis1, Pu.1, Lyl1, Runx1, Scl
EML	Runx1, Tcf7
Erythroid progenitors	Gata1, Gata2, Smad1
MK progenitors	Cbfb, Ring1b, Runx1
Myeloid progenitors	Myb
Pro B cells	Ebf1, Smad1
Myeloerythroid	
MK (megakaryocytes)	Gata1
Macrophages	Cebpα, Cebpβ, P65, Pparg, Pu.1, Stat1
Erythroid	Eto2, Gata1, Ldb1, Mtgr1, Pu.1, Scl
Leukemias	
Leukemia	Notch1
MLL leukemia	Af9
T cell leukemia	RbpJ
T-ALL	Notch1
MEL	Cmyb, Cmyc, Chd2, Gata1, JunD, MafK, Max, Mxi1, NelfE, Scl, Smc3, Tbp, Usf2
